# Lupus Miliaris Disseminatus Faciei as a Diagnostic and Therapeutic Challenge in Facial Dermatoses

**DOI:** 10.7759/cureus.113976

**Published:** 2026-08-04

**Authors:** Ahmed Alshbiti, Syrine Hamada, Benzekri Laila

**Affiliations:** 1 Dermatology, Ibn Sina University Hospital Center, Mohammed V University, Rabat, MAR

**Keywords:** dermatology case report, dermoscopy findings, facial dermatosis, granulomatous dermatitis, lupus miliaris disseminatus faciei

## Abstract

Lupus miliaris disseminatus faciei (LMDF) is a rare inflammatory granulomatous dermatosis of unknown etiology, primarily affecting young adults. It presents as a symmetric papular eruption, predominantly on the mid-facial region, often leading to unsightly depressed scars. We report the case of a 28-year-old woman with persistent facial papules evolving into atrophic scars. Dermoscopy showed orange-brown globules, fine linear vessels, and scarred areas. Histology revealed epithelioid granulomas with fibrinoid necrosis and a lymphocytic infiltrate. The patient was treated with isotretinoin (20 mg/day for nine months) with partial improvement. Management of LMDF remains challenging due to the absence of standardized treatments. Therapeutic options include dapsone, oral corticosteroids, isotretinoin, biologic agents, and laser therapy. This case highlights the importance of early diagnosis and individualized treatment to minimize scarring and psychological impact.

## Introduction

Lupus miliaris disseminatus faciei (LMDF), first described by Tilbury Fox, is a rare inflammatory granulomatous dermatosis primarily observed in adult women. Its etiology remains poorly understood, and no standardized treatment exists. The main characteristics include a papular eruption, often in the mid-facial region, potentially affecting the eyelids and perioral area. The disease usually follows a spontaneously resolving course but often leaves unsightly, depressed, round scars, making early recognition and appropriate management clinically important [1,2]. Histology reveals epithelioid and giant-cell granulomas with central necrosis and a lymphocytic infiltrate [3,4].

The diagnosis of LMDF is challenging because it clinically and/or histologically overlaps with several other granulomatous facial dermatoses, including cutaneous tuberculosis (tuberculides), sarcoidosis, granulomatous rosacea, and acneiform eruptions. Unlike cutaneous tuberculosis, LMDF is not associated with mycobacterial infection, and tuberculin/interferon-gamma release assays are typically negative. It differs from sarcoidosis by the presence of caseation-like central necrosis within granulomas and the absence of systemic involvement. Granulomatous rosacea and acneiform eruptions, meanwhile, generally lack the characteristic central necrosis and tend to follow a more chronic, relapsing course rather than the self-limited evolution typical of LMDF. These distinguishing features support LMDF as a distinct clinicopathological entity rather than a variant of these other conditions.

Treatment remains challenging due to the lack of controlled studies and the incomplete response to various proposed therapies [2]. Through this report, we aim to illustrate the diagnostic reasoning and therapeutic approach in a case of LMDF, highlighting the differential diagnosis process and management with isotretinoin, in order to raise awareness of this underrecognized condition among non-specialist readers.

## Case presentation

A 28-year-old female patient, with no relevant personal or family medical history and no known systemic disease, presented with a slowly progressive papular facial eruption evolving over six months, without associated functional signs, such as pruritus, pain, or burning sensation. The patient reported no fever, arthralgia, or other systemic symptoms. She had previously received oral cyclines for four months without improvement. The lesions consisted of a mix of active papules and older, resolving lesions, the latter leaving depressed scars with significant psychological and cosmetic impact.

Clinical examination revealed multiple reddish to brownish papules, 1-3 mm in diameter, smooth-surfaced, non-tender, and non-fluctuant, associated with depressed round scars, symmetrically distributed over the forehead, eyelids, nose, cheeks, perioral region, and chin (Figure 1). No comedones, pustules, or telangiectasia typical of acne or rosacea were observed. No mucosal, ocular, or lymph node involvement was noted, and systemic examination was unremarkable.

**Figure 1:**
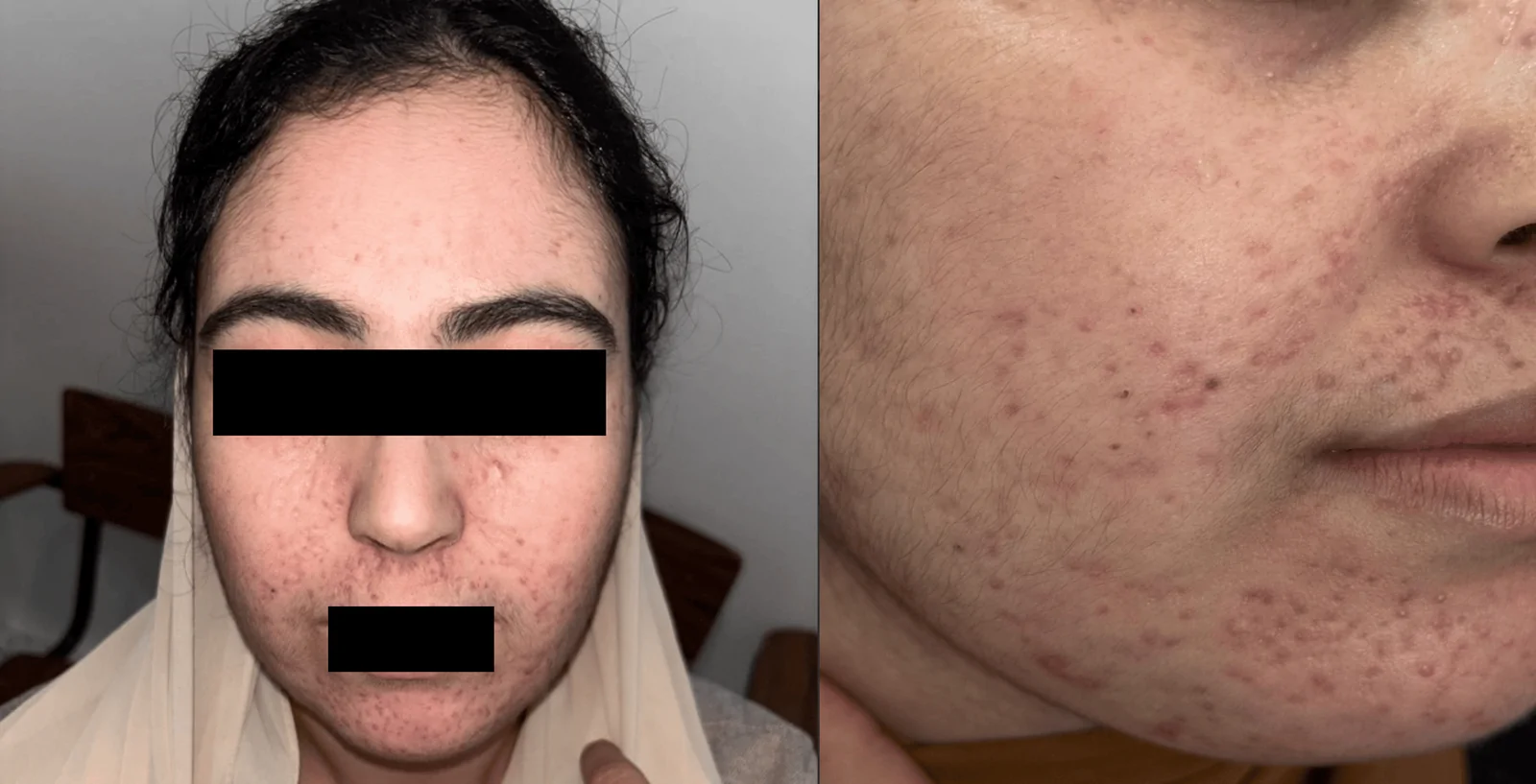
Clinical presentation of lupus miliaris disseminatus faciei. Frontal and lateral views of the face showing multiple erythematous-to-brownish papules, each measuring a few millimeters in diameter, symmetrically distributed over the forehead and cheeks, with atrophic depressed scars from previously resolved lesions.

Given the granulomatous facial presentation, the differential diagnoses of lupus vulgaris, sarcoidosis, granulomatous rosacea, acneiform eruptions, deep fungal infection, and foreign body granulomatous reaction were considered. Chest radiography and a tuberculin skin test/interferon-gamma release assay were performed and were unremarkable, arguing against cutaneous tuberculosis. Serum angiotensin-converting enzyme level and chest imaging showed no evidence of systemic involvement, making sarcoidosis unlikely. Mycological and microbiological cultures were negative, excluding an infectious or deep fungal etiology.

Dermoscopic examination showed brown-orange globules surrounded by mild erythema, fine punctate and linear vessels, white scarred areas, and mild scaling (Figure 2), a pattern consistent with previously reported dermoscopic features of LMDF.

**Figure 2:**
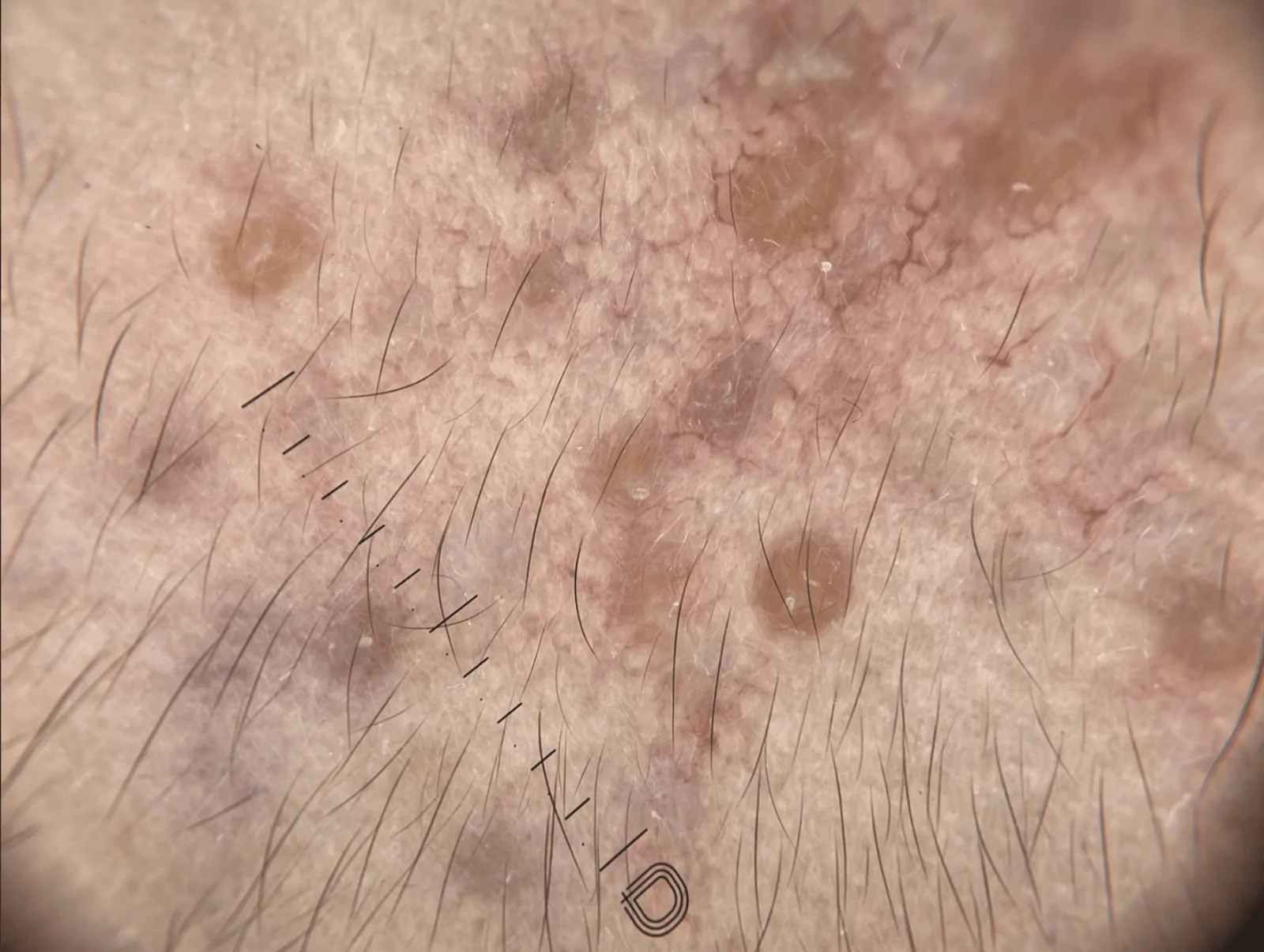
Dermoscopic presentation of lupus miliaris disseminatus faciei, showing round-to-oval orange-brown structureless areas corresponding to the underlying dermal granulomatous infiltrate, set against a pale pink-white background, with fine vellus hairs overlying the lesions.

Histological examination revealed a preserved epidermal layer with a thin orthokeratotic layer. The superficial and mid-reticular dermis showed Periodic acid-Schiff (PAS)-positive fibrinoid necrosis with peripheral fibrosis, epithelioid cells without visible giant cells, and a crown of small lymphocytes, without evident caseating necrosis (Figure 3). Alcian blue staining showed no mucin deposits within the necrotic focus, and no microorganisms were identified on special stains (PAS and Ziehl-Neelsen), further supporting the diagnosis of LMDF over tuberculosis or a fungal granulomatous process.

**Figure 3:**
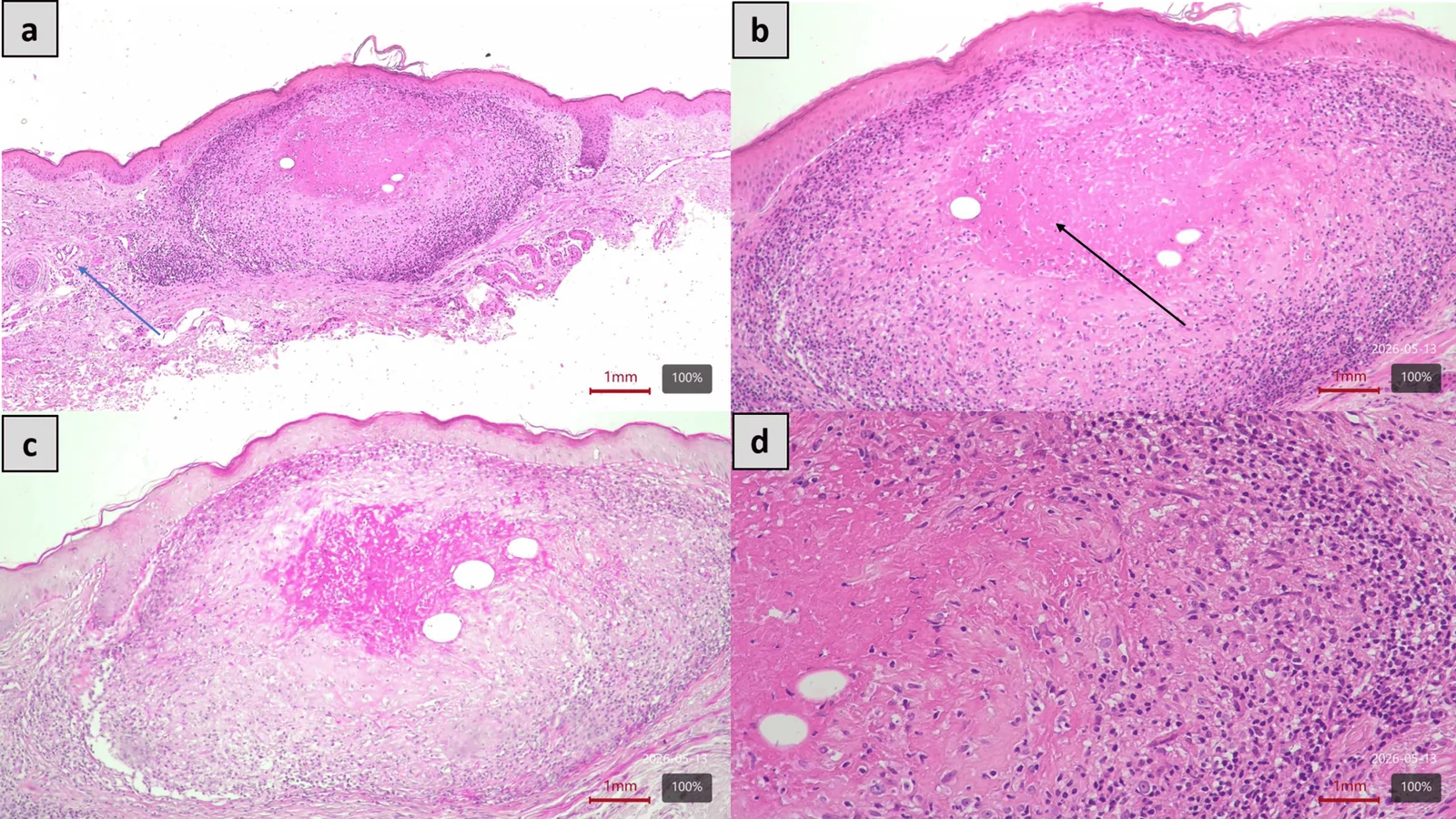
Histopathological findings of lupus miliaris disseminatus faciei. (a) Low-power view showing a well-circumscribed dermal nodular focus of necrosis beneath a preserved epidermis, with an adjacent follicular structure (blue arrow), supporting a follicular-based pathogenesis. (b) Higher-magnification view of the same focus, showing central fibrinoid necrosis (black arrow) surrounded by a dense rim of epithelioid histiocytes and lymphocytes. (c) PAS staining of the same focus highlighting the PAS-positive fibrinoid necrotic material. (d) High-power view of the periphery of the necrotic focus, demonstrating epithelioid histiocytes and a dense lymphocytic infiltrate, without evidence of multinucleated giant cells. Hematoxylin and eosin staining, panels A, B, and D; periodic acid-Schiff staining, panel C; scale bar = 1 mm

Based on the correlation of clinical, dermoscopic, and histopathological findings, and after exclusion of the main differential diagnoses, a diagnosis of LMDF was retained.

The patient was started on oral isotretinoin at 20 mg/day, a dose chosen to balance therapeutic efficacy with tolerability given the chronic nature of LMDF and in line with prior case reports describing benefit from low-dose isotretinoin in this condition [5-7]. Alternative options, including tetracyclines, dapsone, and corticosteroids, were discussed, but isotretinoin was preferred because of its low iatrogenicity. The patient was monitored monthly with clinical examination, and laboratory work-up, including liver function tests and lipid profile, was performed at baseline and monthly thereafter, with no significant abnormalities reported. Treatment was continued for nine months, during which the patient tolerated the treatment well, with only minor mucocutaneous dryness.

At the end of the nine-month course, partial clinical improvement was observed, with a reduction in the number of active papules and in erythematous scarring, and no new lesions during the last three months of treatment. Serial clinical photography was performed at baseline and after nine months to document treatment response (Figure 4). The patient reported improved psychological well-being related to the reduction in active lesions, although residual depressed scarring persisted. The patient was followed for nine months after treatment discontinuation, with no recurrence observed during this period.

**Figure 4:**
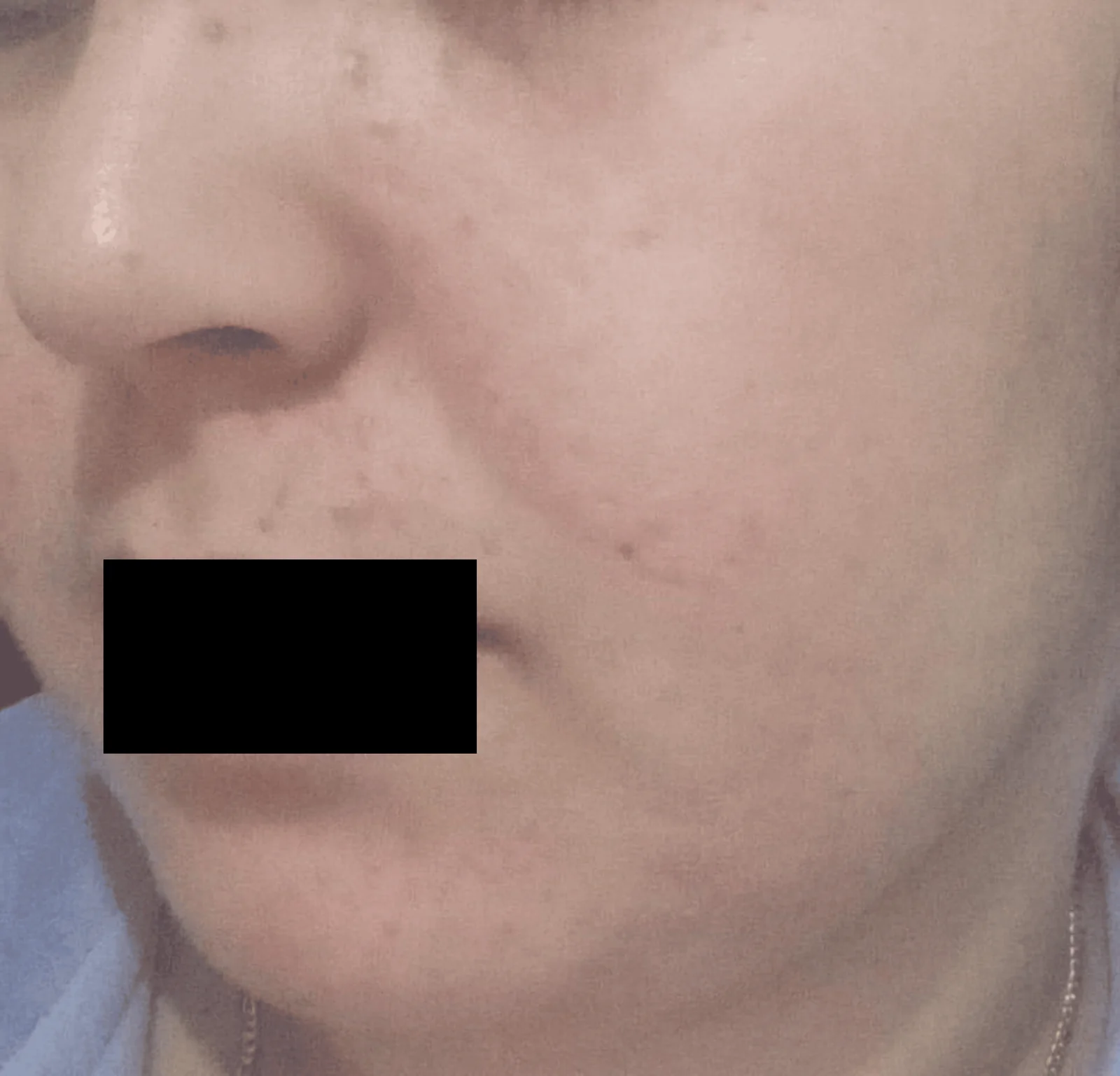
Clinical photographs of the patient at baseline and after nine months of treatment with oral isotretinoin (20 mg/day), showing a reduction in the number of active papules and erythematous scars, with no new lesions during the last three months of therapy, although residual depressed scarring persisted.

Written informed consent was obtained from the patient for the publication of this case and the accompanying clinical images. According to institutional policy, ethics committee approval is required for case series but is not mandatory for single case reports; therefore, no formal ethics committee approval was sought for the present report. The patient was managed at the Department of Dermatology, Ibn Sina University Hospital Center, Rabat, Morocco.

## Discussion

LMDF is a distinct entity that does not correspond to acne, tuberculosis, sarcoidosis, or rosacea. It is a non-infectious granulomatosis despite the presence of giant-cell epithelioid granulomas with caseating necrosis. Its unknown etiology primarily affects young adults.

Clinically, it presents as a papular eruption, rarely pustular, with rounded summits, symmetrically distributed on the face, especially in the mid-facial region, lower eyelids, and upper lip, evolving into deep, round scars. Some studies have reported rarer extra-facial locations, including the ears, neck, back, scalp, legs, axillae, umbilical region, chest, penis, scrotum, wrists, forearms, hips, and buttocks [1,3,4,8,9].

Dermoscopy reveals orange-yellow granulomas indicative of granulomatous inflammation, white or scarred areas associated with fibrosis or tissue remodeling, slight scaling, punctate or fine linear vessels, and follicular plugs resulting from hyperkeratosis and pressure from adjacent granulomas [10].

Histologically, regardless of lesion location, LMDF is characterized by giant-cell and epithelioid granulomas typically centered around caseating necrosis. This feature differentiates it from tuberculosis, which is unresponsive to antitubercular treatment. Some cases exhibit epithelioid granulomas without central necrosis, resembling sarcoidosis or foreign-body granulomas, or even abscesses. Rare cases present with non-specific, non-granulomatous inflammatory infiltrates.

The histogenesis involves follicular aggression with secondary granulomatous reaction, although the target antigen remains undefined. Spontaneous regression is observed in two to four years in most cases.

The management of LMDF is challenging due to the lack of controlled studies. Table 1 summarizes the main treatment options reported in the literature, including dapsone, oral corticosteroids, intralesional triamcinolone, doxycycline, and isotretinoin, with generally favorable but heterogeneous outcomes [2,5,11-14]. However, these data derive from a limited number of cases per treatment and the absence of controlled or comparative trials, which limits the strength of any therapeutic recommendation and underscores the largely empirical nature of current treatment choices, including in our case.

**Table 1:** Summary of previously reported treatments for lupus miliaris disseminatus faciei (LMDF), including dapsone, oral corticosteroids, intralesional triamcinolone, doxycycline, and isotretinoin, with variable outcomes reported across small case series and isolated case reports. Source: Refs [2,5,11-14]

Author	Number of Cases	Treatment	Dose	Outcome
Kumano et al. [11]	12	Dapsone	25-75 mg/day	Recovery in 9 months
Al-Mutairi [2]	8	Dapsone	100 mg/day	Excellent to good response in 7/8 cases
Uesugi et al. [12]	4	Oral corticosteroids	10 mg/day for 2 weeks, then 5 mg/day for 3 months	Recovery in 3/4 cases
Tokunaga et al. [14]	1	Triamcinolone injections	40 mg/month	Excellent response after 3 months
Goh et al. [13]	1	Doxycycline	200 mg/day	Good response
Berbis & Privat [5]	1	Isotretinoin	1 mg/kg/day	Good response

Among these options, dapsone is considered a first-line treatment due to its anti-inflammatory and antineutrophilic properties, showing excellent to good therapeutic responses in most patients [2,11]. It inhibits folic acid synthesis in infectious organisms, lysosomal enzyme release, neutrophil chemotaxis, iodide conversion, lymphocyte transformation, and the alternative complement pathway while enhancing the anti-inflammatory action of corticosteroids.

Oral corticosteroids have been used successfully, particularly in antibiotic-resistant cases, with intralesional triamcinolone injections also showing efficacy. Topical corticosteroids, however, are ineffective [2,12,14,15].

Antibiotics have been used without significant benefit. Antitubercular drugs, paradoxically, are ineffective. Some reports suggest that doxycycline may be beneficial, but tetracycline and minocycline have not demonstrated efficacy [13].

Isotretinoin has been used successfully, with some studies reporting good responses at 1 mg/kg/day. Its mechanism of action in LMDF may relate to its effects on the immune system and sebaceous apparatus [5-7].

Combination therapies, such as dapsone with prednisone or topical tacrolimus (0.03%), have yielded excellent outcomes [2]. Biologic therapies, including adalimumab, have also shown promising results [16].

Laser treatments, including the 1450 nm diode laser and 1565 nm non-ablative fractional laser, have demonstrated efficacy. For scar management, CO_2_ laser combined with 100% TCA has shown success [17-19]. Early corticosteroid treatment at low doses may prevent scarring [20].

## Conclusions

LMDF is a rare but distinct granulomatous entity, typically following a self-limiting course but often resulting in unsightly, disfiguring scars. No treatment has proven universally effective, likely reflecting the still unclear etiopathogenesis of the disease and the absence of standardized, evidence-based management protocols. In our patient, isotretinoin at 20 mg/day for nine months resulted in partial clinical improvement, in line with previous reports of variable and incomplete treatment responses in LMDF. Older, well-established lesions appear more resistant to treatment, underscoring the importance of early recognition to limit scarring. Longer follow-up and standardized outcome assessment in future cases are needed to better define the natural history of LMDF and to establish more reliable therapeutic strategies.
